# Quantitative Imaging with a Mobile Phone Microscope

**DOI:** 10.1371/journal.pone.0096906

**Published:** 2014-05-13

**Authors:** Arunan Skandarajah, Clay D. Reber, Neil A. Switz, Daniel A. Fletcher

**Affiliations:** 1 Department of Bioengineering, University of California, Berkeley, Berkeley, California, United States of America; 2 Biophysics Graduate Group, University of California, Berkeley, Berkeley, California, United States of America; 3 Physical Biosciences Division, Lawrence Berkeley National Laboratory, Berkeley, California, United States of America; University of Cambridge, United Kingdom

## Abstract

Use of optical imaging for medical and scientific applications requires accurate quantification of features such as object size, color, and brightness. High pixel density cameras available on modern mobile phones have made photography simple and convenient for consumer applications; however, the camera hardware and software that enables this simplicity can present a barrier to accurate quantification of image data. This issue is exacerbated by automated settings, proprietary image processing algorithms, rapid phone evolution, and the diversity of manufacturers. If mobile phone cameras are to live up to their potential to increase access to healthcare in low-resource settings, limitations of mobile phone–based imaging must be fully understood and addressed with procedures that minimize their effects on image quantification. Here we focus on microscopic optical imaging using a custom mobile phone microscope that is compatible with phones from multiple manufacturers. We demonstrate that quantitative microscopy with micron-scale spatial resolution can be carried out with multiple phones and that image linearity, distortion, and color can be corrected as needed. Using all versions of the iPhone and a selection of Android phones released between 2007 and 2012, we show that phones with greater than 5 MP are capable of nearly diffraction-limited resolution over a broad range of magnifications, including those relevant for single cell imaging. We find that automatic focus, exposure, and color gain standard on mobile phones can degrade image resolution and reduce accuracy of color capture if uncorrected, and we devise procedures to avoid these barriers to quantitative imaging. By accommodating the differences between mobile phone cameras and the scientific cameras, mobile phone microscopes can be reliably used to increase access to quantitative imaging for a variety of medical and scientific applications.

## Introduction

Mobile phones are becoming an important part of the healthcare system, with use of mobile phone cameras to capture clinically relevant images growing rapidly in recent years. For example, doctors have demonstrated new ways to interact with and record medical image data in specialties such as dermatology and neurosurgery [1]–[3]. To address healthcare needs in low-resource regions, researchers have also shown that mobile phones can bring traditional diagnostic assays to inadequately served populations [4]–[6]. Several groups have developed small footprint custom devices that take advantage of the imaging, connectivity, and processing capabilities of the phone for applications including microscopic imaging [7], [8], holographic imaging [9], label-free spectroscopy [10], and image-based quantification of diagnostic tests [11], [12].

While mobile phones have the potential to enable image-based diagnosis outside of traditional clinics and even replace more expensive instruments, the quality of images taken with mobile phones – and the variation in image quality due to different phones – remains an issue of concern. Unlike mobile phone cameras, specialized scientific cameras used for quantitative optical imaging applications in medicine allow independent control of parameters such as exposure time and color balance, and they provide access to raw image data free from post-processing. With these cameras, imaging parameters can be carefully chosen and maintained so that clinicians can obtain accurate and repeatable information needed for diagnostic decision making. For example, diagnosis of malaria species from images of blood smears requires micron-scale resolution of parasite shape [13], while the speed of diagnosing malignancy on breast biopsy slides has been shown to improve with consistent color information [14].

Mobile phone cameras, on the other hand, permit limited control of camera parameters and often allow no control over post-processing done on the image before viewing or transmission. Exposure time and electronic gain are automatically adjusted on most phones to prevent over or under-exposure, but this can cause variations in brightness and color response within or between specimens, making comparisons across images by human viewers or by automated analysis software difficult. Furthermore, demosaicing, noise reduction, edge sharpening, and image compression are automatically carried out on most mobile phone images without input from the user [15]. Implementation of these algorithms across phones varies significantly [16], [17], is not typically lossless, and may not represent an optimal use of the available pixel information [18], further complicating attempts to quantify and compare images.

These barriers to quantitative imaging raise important questions about mobile phone microscopes: are mobile phones in their current form suitable for diagnostic applications, and do lower-cost optical components used in mobile phone microscopes compared to scientific microscopes ultimately limit their capabilities? Initial efforts to use mobile phone cameras for both medical and scientific applications highlighted these concerns, with some studies concluding that mobile phones are inadequate for pathology [1] or limited in their image quality [19]. However, other work has shown that a full-size microscope equipped with a phone camera can qualitatively capture relevant features of malaria and TB [6]. These opposing conclusions, along with recent advancements in the hardware and software of mobile phone cameras, point to the need for a detailed analysis of quantitative imaging with a mobile phone-based microscope.

In this work, we systematically characterize the image quality achieved by mobile phone cameras when used as part of a mobile phone microscope. We quantify the effect of iPhone and Android phones released in the period of 2007–2012 on the spatial resolution of images taken with a custom mobile microscope, known as CellScope, outfitted with an adaptor suitable for use with multiple phone types. We also examine additional characteristics of the mobile phone microscopy system including brightness uniformity across the field of view, degree of image distortion, and nonlinear encoding of pixel intensity, which can be corrected through a gamma transformation. We then address barriers to the use of mobile phones for quantitative imaging caused by the automatic camera parameter adjustments implemented by most phones. We demonstrate artifacts that result in nonlinear response to input signals, variation in spectral sensitivity, and changes in effective magnification – all of which can compromise diagnostic imaging – and we outline approaches to achieve more reproducible and consistent imaging. The results of our study indicate that mobile phone microscopes can indeed provide reliable and repeatable images suitable for diagnostic use.

## Results

### System Design and Mobile Phone Integration

**Figure 1:**
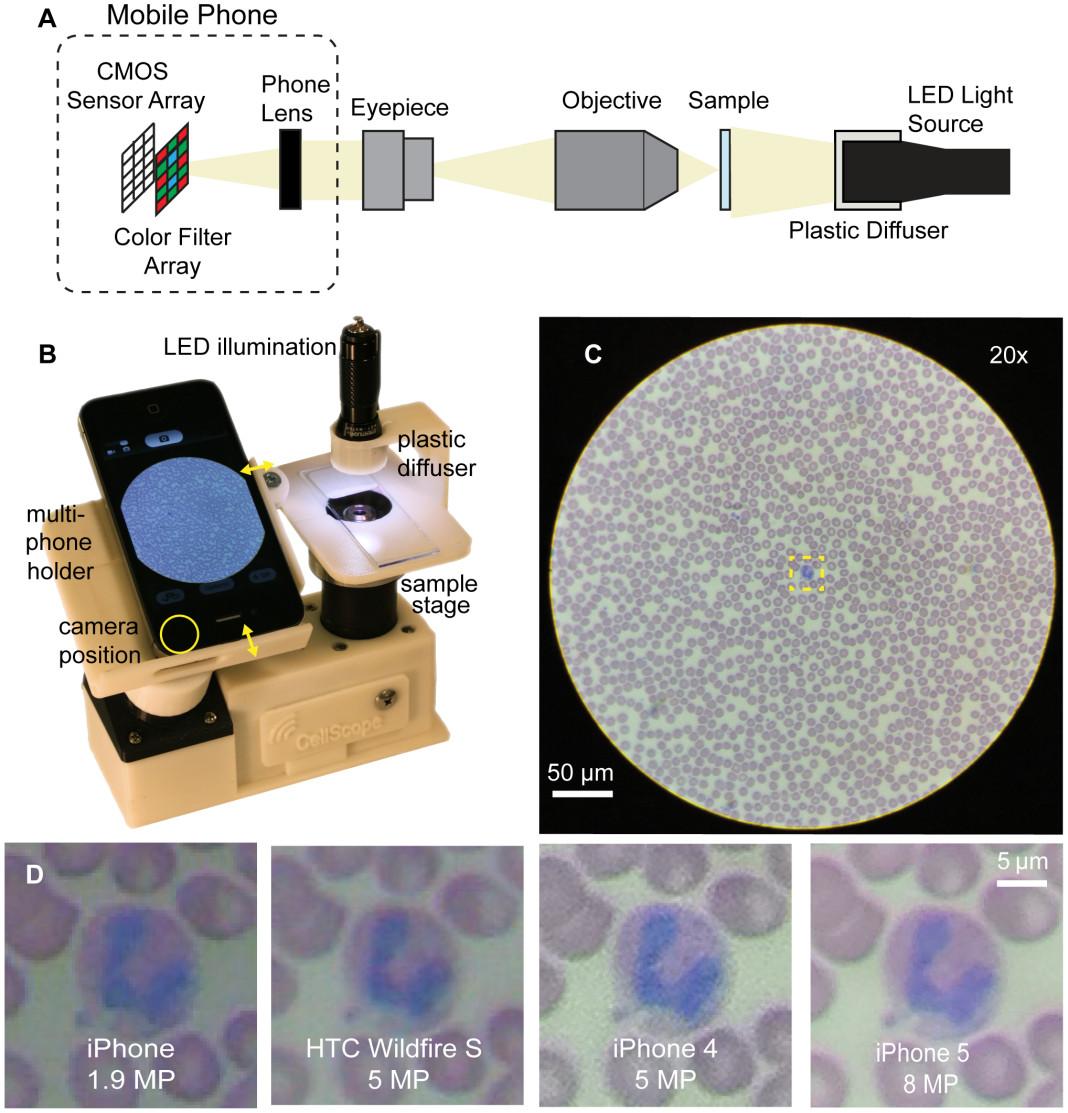
A multi-phone mobile microscope. **A** Diagram of the magnifying optics and illumination added to a mobile phone to create a transmission light microscope. **B** Prototype of a field-ready mobile microscope – the CellScope – that has a folded optical path for compactness and is equipped with a multi-phone holder and iPhone 4. Phone-specific variants have been evaluated on five continents for various applications. **C** A Wright stained blood smear taken on the mobile microscope with an iPhone 4 and 20×/0.4 NA objective showing the inscribed field of view captured by the device. **D** Enlarged images of the small region of interest in **C** containing a granulocyte and red blood cells taken with four different mobile phones. The images demonstrate resolution, color, and brightness differences among phones.

We utilize a finite conjugate transmission microscope consisting of an achromatic objective coupled to a mobile-phone camera using a 20x widefield eyepiece, as in our earlier work [7], with the addition of folding mirrors to create a more compact and stable design (Figure 1). To date, variants of this basic CellScope mobile phone microscope with phone-specific adapters have been tested with clinical partners in multiple countries [20]. The optics of the microscope attachment and mobile phone camera module are diagrammed in Figure 1A, and a fully assembled CellScope prototype constructed with a two-axis sliding mechanism for aligning a variety of phones with the microscope attachment is shown in Figure 1B. The optics casing, adaptor for multiple phone types, sample stage, and plastic diffuser are made with a 3D printer from ABS plastic. The optics casing orients and supports the microscope optics while blocking out light that does not pass through the sample and would otherwise reduce contrast in the final image. The phone holder couples the phone to the microscope optics by aligning the phone camera with the eyepiece and setting the distance between the two to position the mobile phone lens at the exit pupil of the eyepiece, thus minimizing vignetting and maximizing the area of the camera sensor filled by the image. The stage provides a surface for mounting the sample slide, and focus is achieved by adjusting the height of the sample stage with a threaded adapter. Because the stage snaps on to the assembly, application-specific stages that permit imaging of a broad range of sample geometries, such as those on slides or in capillaries, can be switched in and out without changes to the optics [20]. A commercially available broad-spectrum LED flashlight powered by field-replaceable watch batteries provides illumination.

The optical design of the microscope attachment yields a circular field of view that is inscribed on the rectangular CMOS camera sensor of the mobile phones, as shown in Figure 1C. The same region of a blood smear showing red blood cells and a granulocyte was centered and imaged across a range of mobile phones coupled to the same mobile phone microscope. While the various phones provide similar morphological information, there are visible differences in resolution, brightness, and color balance (Figure 1D). For example, the pixelated images taken with older mobile phones having lower pixel count sensors – and also typically having less well-corrected camera lenses incorporating fewer optical elements – fail to capture detail around the nucleus of the granulocyte. Independent of this effect, the mean intensity and distribution of colors of the cells and surrounding area vary noticeably across the images. To understand the cause of these differences, we separately investigated spatial resolution, image contrast, and color with standardized samples.

### Achieving High Quality, Sub-Micron Imaging with a Mobile Phone Microscope

We began our evaluation of quantitative imaging with a mobile phone microscope by determining whether images collected with the CellScope mobile phone microscope followed the spatial resolution limits of the microscope optical system itself. Our resolution metric is based on the smallest distinguishable set of bars in a high resolution 1951 US Air Force test target. For most purposes, features smaller than the measured resolution limit do not have sufficient contrast to be useful for human or computer analysis. While there are known limitations of three-bar targets to determining the image quality of a system [21], [22], the method nevertheless represents an intuitive, widely-used, and consistent measure of resolution.

**Figure 2:**
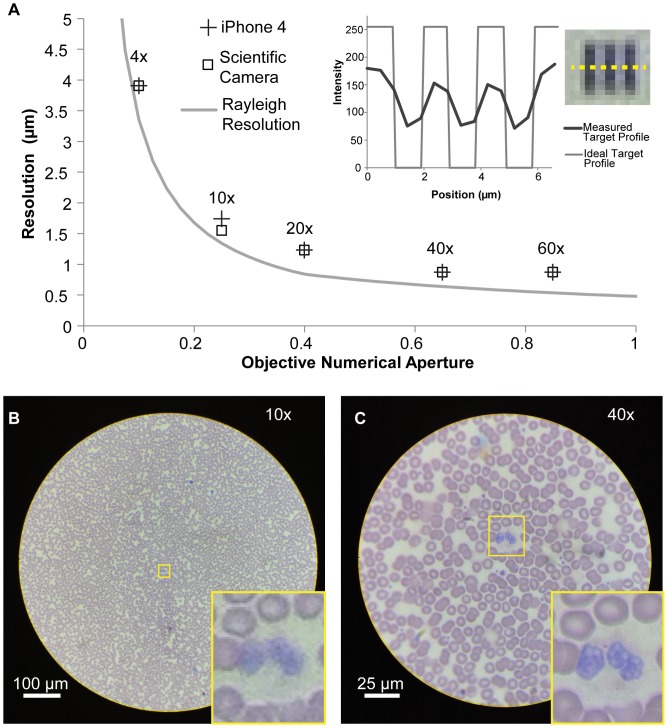
Spatial resolution of mobile phone microscopy is dependent on microscope optics. **A** The resolution that can be captured with a mobile phone microscope approaches that of a scientific camera coupled to the same optics across a range of numerical apertures. Inset shows the measured intensity profile across bars of non-transmitting chrome spaced at 512 line pairs per millimeter and taken with a 10×/0.25 NA objective, as well as the ideal target profile. The Michelson contrast calculated for this example group is 41%, indicating that features with this spacing are resolved. **B** Wright stained blood smear with an inset of a granulocyte and red blood cells taken with a 10×/0.25 NA objective and iPhone 4. **C** Image of the same sample and region of interest taken with a 40×/0.65 NA objective and iPhone 4 showing improved resolution.

To quantify resolution, we measured intensity profiles across labeled sets of three non-transmitting chrome bars oriented vertically and horizontally (Figure 2A, inset). After background subtraction, we then calculated the Michelson contrast [23] for each set of bars. Our resolution determination is based on the smallest elements for which the Michelson contrast for both the vertical and horizontal bars is greater than 10%. Using a mid-tier iPhone 4 (5 MP camera, introduced in 2010), we repeated this for five different objectives with different magnifications and numerical apertures (NA): 4×/0.10 NA, 10×/0.25 NA, 20×/0.40 NA, 40×/0.65 NA, 60×/0.85 NA (Figure 2B). To isolate the role of the sensor and lenses of the mobile phone camera module from the microscope optics in determining image quality, we also coupled the system to a scientific camera using an achromatic lens with a 50 mm focal length, which resulted in digital oversampling of the image (≥4 * Nyquist requirements), eliminating any effects due to undersampling [24] and attendant aliasing by the phone. We find that the iPhone 4 and scientific camera agree well for most objectives, indicating that a 5 megapixel mobile phone with a typical ∼4 mm focal length and f/2.8 lens is capable of capturing the resolution information collected by a representative low-cost microscope system using a 20x eyepiece.

For an ideal imaging system, resolution is expected to scale with the numerical aperture (NA) of the objective and condenser as governed by the Rayleigh criterion for the minimum distinguishable distance between two points, δ = 1.22 λ_0_/(NA_obj_+NA_cond_), where λ_0_ for a broadband white light source is taken as 550 nm, roughly the peak of the human photopic visual response [25], [26]. Since the transmitted-light illumination on the mobile phone microscope is from a simple plastic diffuser rather than a more complicated and expensive Kohler illumination system, the condenser NA is imperfectly defined, but is ∼ 0.4 for our system based on the angle defined by the radius of the plastic diffuser and its distance from the sample. For purposes of determining expected resolution, we approximate the condenser NA as equal to 0.4 when using objectives of NA ≥0.4, and as equal to the objective NA for NA_obj_ <0.4 due to the reasonably flat dependence of resolution on condenser NA for NA_cond_>NA_obj_ [25]. We find that the measured spatial resolution of our mobile phone microscope improves with objective NA, as expected theoretically, but is consistently worse than that based on the Rayleigh criterion (Figure 2A).

We expect that resolution of the mobile phone microscope will be worse than the Rayleigh criterion due to the low price point of the objectives used ($75–200), with a more significant penalty for high NA objectives that are more complex to construct and hence suffer from greater proportional performance degradation at low price points. Consistent with this, we find that increasing objective numerical aperture and magnification from 40×/0.65 NA to 60×/0.85 NA does not yield a measurable increase in microscope resolution, likely due both to insufficient aberration correction and to the reduced resolution improvement expected when the objective NA increases beyond the condenser NA (Figure 2A). In addition to cost, resolution and field of view are both important considerations for diagnostic imaging such as blood smears or sputum samples. The trade-off between resolution and field of view of the 10×/0.25 NA and 40×/0.65 NA objectives for imaging of red blood cells and a granulocyte is shown in Figure 2B and 2C. While measured resolution improves by a factor of two (from 1.74 µm to 0.87 µm), there is a decrease in the diameter of the field of view by a factor of four (from 812 µm to 205 µm). This corresponds to a reduction in imaged area by 16x, the square of the magnification, which can have a large impact on diagnostic sensitivity.

**Figure 3:**
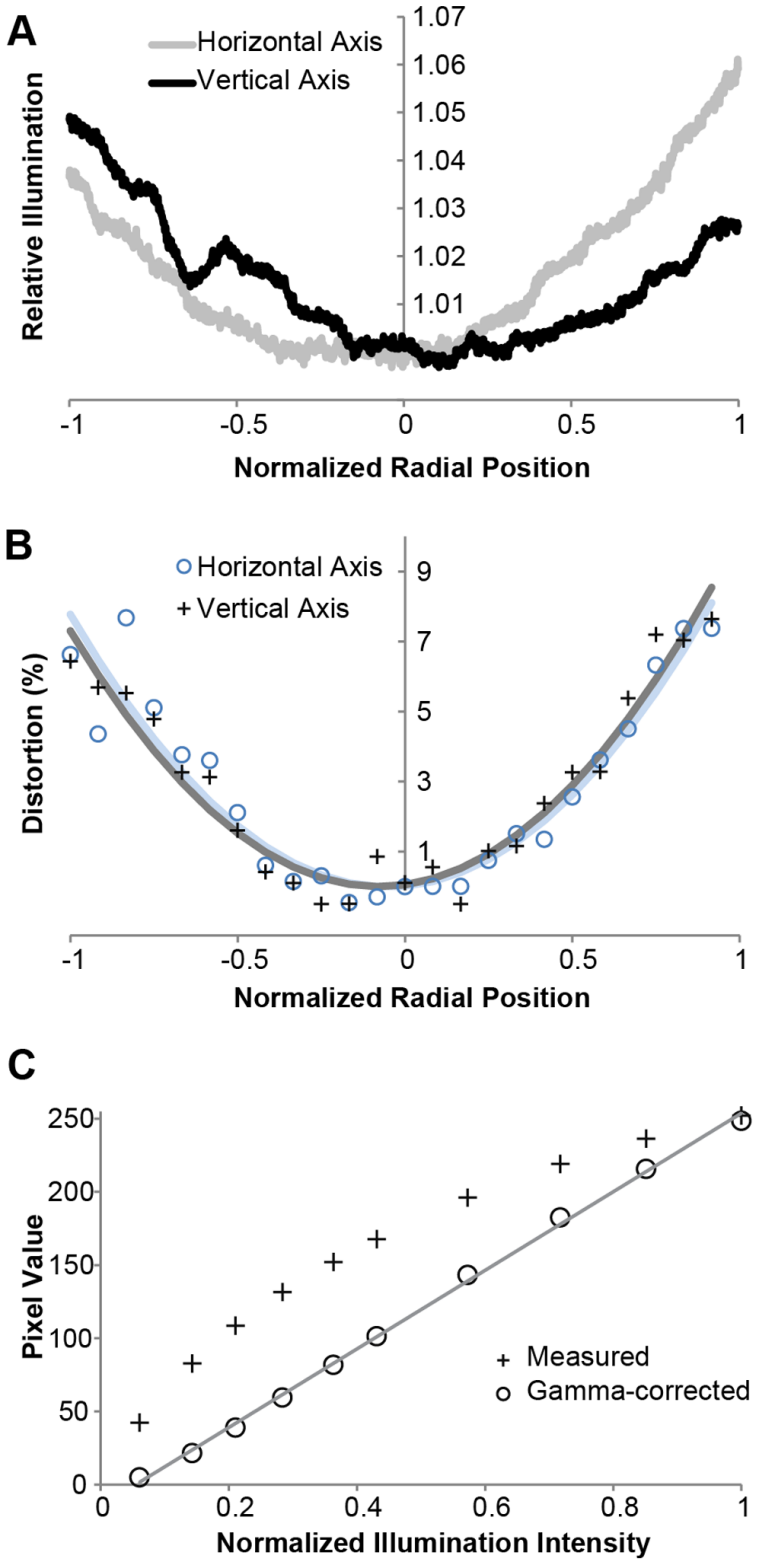
Illumination variation, image distortion, and pixel non-linearity of mobile phone microscopy can be minimized. **A** Variation in illumination across a clear field of view along the horizontal and vertical axes for an LED flashlight source evaluated with a 10×/0.25 NA objective and iPhone 4. **B** Distortion across a field of view, evaluated along a bar target with a 10×/0.25 NA objective and iPhone 4. A parabolic fit is superimposed on the data for both axes. **C** The measured pixel response (**+**) of an iPhone 4 and 10×/0.25 NA objective to changes in illumination intensity is nonlinear but can be corrected for the gamma encoding (**o**) to recover a linear pixel response (R^2^ = 0.999).

Additional properties that affect the quality of imaging with a mobile phone microscope include the variation in measured illumination across a field of view, image distortion, and linearity in the pixel response. We find that the total detected intensity varies by less than 7% across a clear field of view (Figure 3A), due to a combination of non-uniform illumination from the single LED, sensor sensitivity, and vignetting, among other factors [26]. We utilized a regularly spaced line grating to measure the spatial dependence of magnification and find that distortion remains below 8% in the image (Figure 3B). This degree of distortion is reasonable for a system employing a microscope eyepiece, which typically introduces pincushion distortion on the order of 3–12% [26]. Finally, we quantified the pixel response as a function of intensity using a calibrated, spectrally constant light source. We find that the response is monotonic across the 8-bit range but is not linear (Figure 3C). Notably, a linear response to incident intensity can be recovered on a mobile phone by conducting a gamma transformation with an exponent of 2.2 [27], corresponding to the inverse of the gamma encoding algorithm for sRGB images (Figure 3C). While surprising from the perspective of scientific imaging, where intensity in an image is proportional to the recorded pixel value, the mobile phone represents and displays intensities according to an sRGB color space [27]. For applications that rely on the linearity of intensity, such as measuring concentration by absorption or fluorescence, gamma decoding is therefore necessary.

### Quantifying the Impact of Phone Choice on Spatial Resolution

While mobile phone microscopes can capture high quality images, is image resolution dependent on exactly which phone is used? We investigated this issue by comparing the performance of a selection of Apple and Android mobile phones coupled to a 10×/0.25 NA air objective and 20x widefield eyepiece. This combination results in an apparent angular magnification, as compared to viewing the sample by eye from a distance of 25 cm, of 200x prior to the mobile phone lens. The arrangement was chosen for demonstration because it provides sufficient magnification for single cell imaging and is a practical magnification used in diagnostic microscopy where usability and cost limit the utility of higher NA objectives and oil immersion. Phones evaluated in this study are the iPhone, iPhone 3G, iPhone 3GS, iPhone 4, iPhone 4S, iPhone 5, HTC G1, Samsung Galaxy Ace, HTC Wildfire S, and LG Nexus 4.

**Figure 4:**
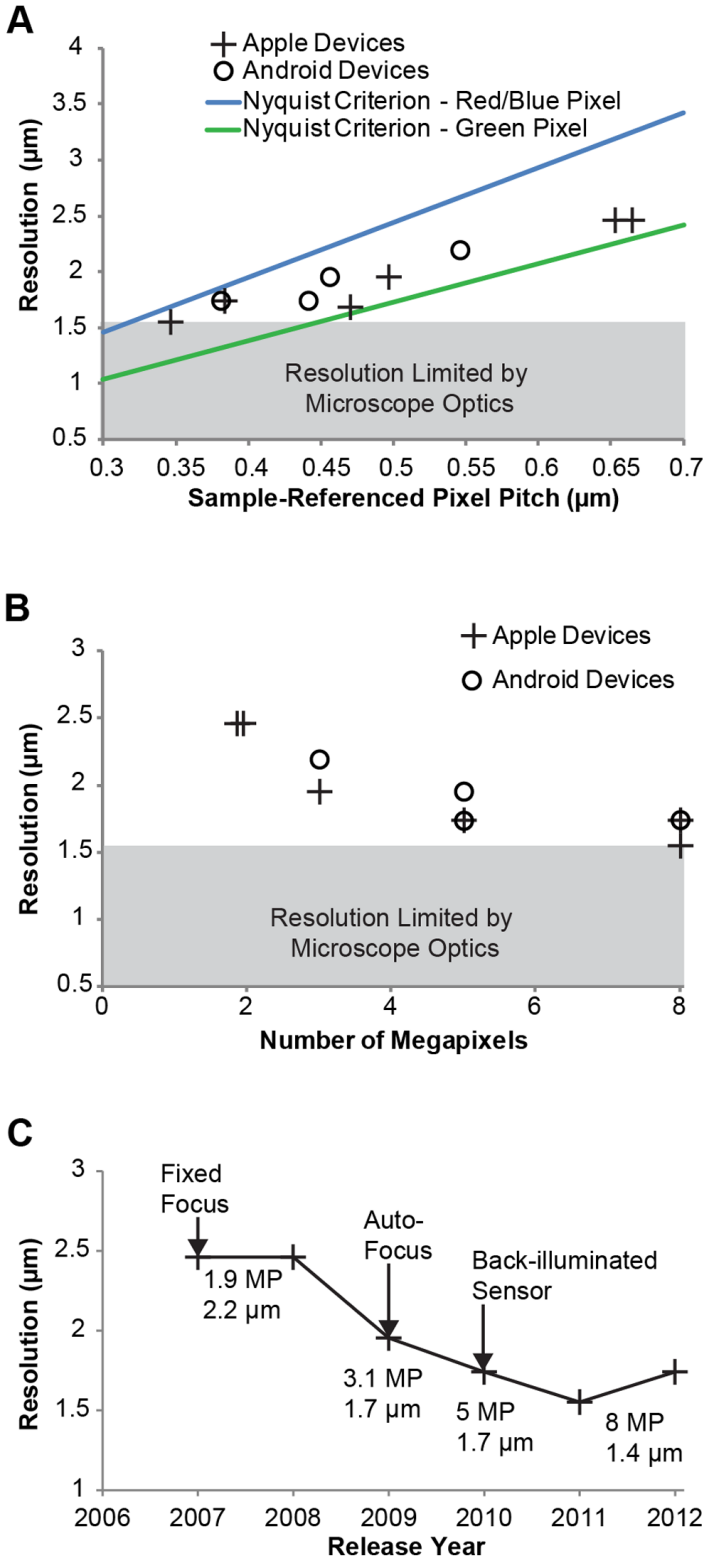
Spatial resolution of mobile phone microscopy has improved with mobile phone advancement. **A** The spatial resolution of mobile phone microscopy with iPhone and Android phones is plotted as a function of the effective pixel size for images taken with a 10×/0.25 NA objective. The theoretical constraints on resolution imposed by pixel spacing on the Bayer color sensor array are plotted along with the empirically determined resolution limit of the underlying microscope optics. **B** The spatial resolution of mobile phone microscopy with the same iPhone and Android phones is plotted as a function of megapixel count. **C** Spatial resolution of the iPhone family of phones is plotted over time, together with the dates of significant camera advancements.

We quantified the spatial resolution of images taken with each phone as a function of sample-referenced sensor pixel pitch (Figure 4A). The sample-referenced (effective) sensor pixel pitch (*p*) is the pixel spacing as imaged from the sensor to the sample, i.e., demagnified by the total optical magnification as determined by calibration with a sample of known size. To understand how the pixel spacing affects the ability of a mobile phone to capture the spatial resolution information collected by the optics, we consider the highest spatial frequency captured by the optical system using incoherent illumination. As with the Rayleigh resolution criterion, this value is proportional to the numerical aperture and inversely proportional to the effective wavelength [25]. Imaging without aliasing requires, per the Nyquist criterion, sampling at twice the maximum spatial frequency *k* captured by the system (*k* = λ/2 NA for fully incoherent illumination) [28] and thus sets an upper limit on the sample-referenced pixel pitch. The effective spacing of the pixels is a function of wavelength, since an overlaid filter array limits each pixel to detecting a subset of the visible spectrum, usually corresponding to one of red, green, or blue color. Since the exact layout of the filter array is proprietary, as is the unique demosaicing algorithm used by each phone [17], we assume a common Bayer Filter Array directly on the sensor. This allows us to plot a range of potential resolutions corresponding to the Nyquist limit, specifically a best case based on the spacing of green pixels (δ_green_ = √2*p* * 2.44) and a worst case based on the sparser red and blue pixels (δ_red,blue_ = 2*p* * 2.44). The maximum resolution is also limited by the information collected by the non-phone microscope optics (Figure 4A, gray region), which was determined for the specific objective and eyepiece used in the measurement using a monochrome CMOS camera configured to oversample the microscope output.

Performance of both Apple and Android phones were found to be similar and dependent primarily on pixel density until the optics of the microscope become limiting. We note that regardless of the manufacturer modern phones with five or more megapixels, typically corresponding to effective pixel spacing between 0.44–0.47µm at the sample plane, are able to capture nearly all of the resolution information collected by the microscope with a 10×/0.25 NA objective and 20x widefield eyepiece (Figure 4B). Considering the performance of only Apple phones over time, the resolution of mobile phone microscopy with iPhones has improved 63% over the past five years and six phone models (Figure 4C), together with increasing pixel number and other technological improvements to the phone camera. While the characterization method described here is general to color images when raw pixel image data is unavailable, the specific 5 megapixel threshold described corresponds to the particular objective and eyepiece combination presented. If pixel counts continue to increase for a constant angular field of view, thus decreasing the sample-referenced pixel spacing, the microscope optics can be revised to take advantage of the greater total information content in a single image (known as the space-bandwidth product [29]). This increase in pixel count could be used to improve resolution for a given field of view, for example by using a more expensive objective that is either better-corrected or has a higher numerical aperture at the same magnification. Alternately, the increase in pixel count could be used to increase field of view for a given resolution, for example by using a lower magnification and less-expensive eyepiece.

### Accounting for the Effects of Phone Automation on Image Capture

**Figure 5:**
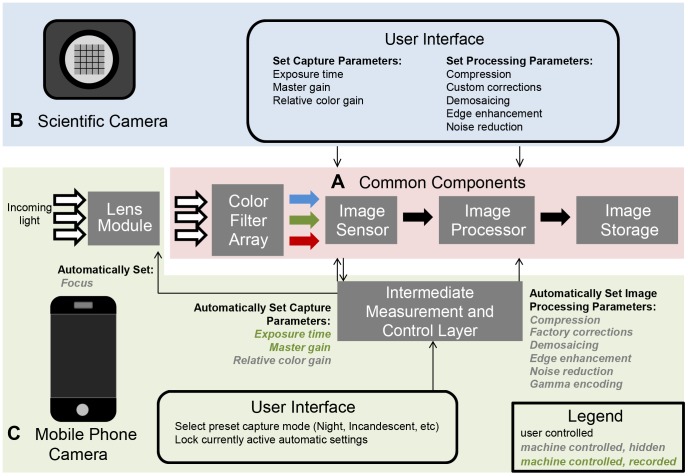
Mobile phones differ from scientific cameras in selection of image capture and processing parameters. **A** Common core hardware components underlie the capture process of both mobile phone cameras and scientific cameras. **B** The capture and processing parameters are set directly through the user interface of a scientific camera. **C** On mobile phones, an intermediate layer assesses the view of the camera in real-time and modifies image acquisition. This simplifies the user interface for traditional point-and-shoot photography but sacrifices the control desired by a scientific user.

**Figure 6:**
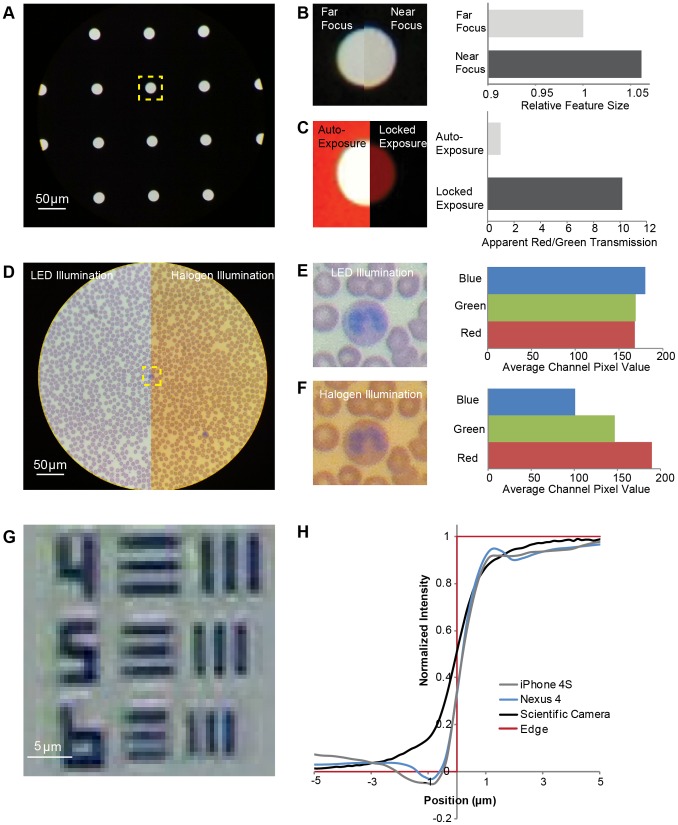
Phone automation during image capture and storage degrades feature and color information. **A** High-contrast image of an array of 20 µm diameter pinholes in chrome taken with an iPhone 4 and 20×/0.40 NA objective. **B** Phone auto-focus changes effective magnification for samples held at different distances from the objective lens, resulting in changes to apparent feature size. **C** Saturation in individual color channels due to auto-exposure and gain causes loss of color contrast in sparse, bright samples. **D** Built-in white-balance of the iPhone 4 is insufficient to overcome variable illumination conditions, resulting in a shift of the apparent color profile of samples such as this blood smear, as quantified in **E** and **F** for a region of interest illuminated with a white LED or halogen lamp, respectively. **G** Sharpening algorithms cause patterns of artificial ringing and haloing around high contrast structures such as this chrome-on-glass resolution target. **H** Intensity profiles at edges show phone-specific deviations from the expected theoretical monotonic profile obtained from an incoherently illuminated sample using a scientific camera and no image processing. The measured profiles are normalized, with 0 corresponding to the intensity in chrome far from an edge and 1 corresponding to the intensity in a clear region far from an edge.

**Figure 7:**
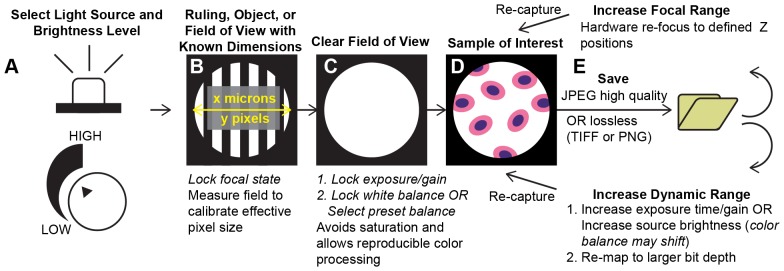
Proposed steps to enable quantitative, reproducible imaging with a mobile phone microscope. **A** Standardize illumination source and brightness. **B** Set focal state on a field with known dimensions or features. **C** Set exposure and gain using a clear field of view. Use this field to set or select a white balance state; may require resetting exposure and gain to ensure changes in white balance do not result in saturation of a color channel. **D** Acquire images of samples while keeping capture settings constant. **E** Information content can be preserved by selecting lossless or high quality compression settings. In addition, multiple images can be used to record additional z planes or expand the effective dynamic range of the image. While many of the features required to implement these steps are not directly accessible in the default camera, they are built into commonly available third-party camera applications or can be incorporated into custom applications.

In addition to the need for sufficient spatial resolution, microscope images must have appropriate contrast and consistent colors to be useful for medical diagnostic applications. Though both scientific cameras and mobile phone cameras use common steps to capture color images (Figure 5A), there are significant differences between cameras that impact image quantification. Scientific users are accustomed to being able to set capture parameters independently and consistently across a set of observations (Figure 5B). In contrast, automated algorithms control focus, exposure, color balance, and image processing in modern mobile phones (Figure 5C), significantly altering image appearance from the raw intensity values. This is further complicated by the default camera application, which prioritizes a simple image capture process and high perceived sharpness over the reproducibility and detailed control desired by scientific users. However, we find that image artifacts caused by automated algorithms can be minimized through a set of corrective procedures implemented as part of the mobile phone microscopy workflow. While details of the algorithms underlying the automatic adjustments carried out by the phone are discussed elsewhere [16], [30]–[34], the consequences (Figure 6) and methods (Figure 7) for overcoming them are outlined here.

#### Magnification is modified by automatic focus

Accurate feature size is often essential for measuring and comparing objects for identification and diagnosis. The camera of a mobile phone automatically adjusts focus in traditional photo capture mode by altering the lens focal length (Figure 5C) and thus the optical magnification. This is also true when mobile phones are used for microscopy, where the auto-focus can change the total magnification and therefore size of objects in an image despite unchanged microscope optics. To demonstrate the role of this auto-focus mechanism, we imaged an array of pinholes in a chrome-on-glass mask (Figure 6A). The auto-focus motion can be useful for accommodating small displacements in the sample plane but is insufficient to fully eliminate the need for a stage focus mechanism on the microscope attachment. This automatic adjustment does however result in changing the effective magnification, thereby changing the measured feature size of a microscope image by as much as 6% (Figure 6B). While this effect is not significant for tasks such as counting blood cells, applications that require more quantitative information on feature size could be impacted by changes in the image scale due to autofocus. For example, monitoring lung pathology requires identifying changes in cell length and area as a function of disease state [35]. To avoid this issue, camera software must be used to fix the focus state and eliminate autofocus prior to image size calibration (Figure 7B).

#### Color capture is impacted by automatic exposure and channel gain

Proper collection of color information is a high priority in the design of imaging pipelines [16], [36] and integral to identification of objects in diagnostic medical imaging applications [37], [38]. Staining dyes transform properties of the sample correlated to pathology into distinct color changes that pathologists, and more recently algorithms [39], are trained to recognize. While sample preparation itself affects color in the final sample, groups conducting cytology-based evaluations have invested significant effort into developing and validating workflows to consistently capture and properly display color information in images [38]. Humans perceive the color of objects to stay approximately constant across illumination conditions with different spectral compositions [40], but electronic sensors have defined wavelength-dependent sensitivity. With a fully configurable camera, users can work with a known light source and apply standard settings to avoid this wavelength dependence, enabling reproducible image capture. Mobile phone cameras, however, have a more opaque control scheme for color data that involves automatic exposure and color channel gain (Figure 5C). This automation, as well as additional software that attempts to mimic human perception, can significantly and unintentionally alter the reported spectral information in the image [36].

For images of samples that do not meet pre-set minimum levels of average brightness [16], the default behavior of mobile phones is to adjust electronic gain and exposure time to meet those minimum brightness levels, which has a significant effect on captured color information. To quantify this effect, we placed a red plastic film (Figure 6C) above the pinhole array discussed previously; such an array is representative of punctate samples in both dark-field and fluorescence. This type of scene, as previously noted in the case of video microscopy [41], triggers excessive gain and exposure time as the auto-exposure algorithm attempts to achieve a moderate average image intensity. The result is loss of color contrast as the apparent ratio of red-to-green light transmission through the pinholes changes from 10∶1 to 1∶1 as different color channels saturate (Figure 6C).

Color information can also be compromised by the phone’s automatic white balancing processes and the properties of the light source. Automatic white balancing adjusts the gains of the three channels independently to meet pre-specified and often proprietary criteria about the color composition of the scene (Figure 5C). This process results in different effective sensitivities for each of the three ranges of wavelengths corresponding to the red, green, and blue components of the color filter array. Changes in the spectral composition of the light source are often more complex than can be corrected for by this type of three-parameter sensitivity adjustment (Figure 6D–F). This means that acquiring color information reproducibly with a mobile phone camera is best accomplished with a consistent light source (Figure 7A), as is the case for a standard clinical microscope with a user-configurable scientific camera. When color is used as a contrast mechanism to enable cell counting or morphology evaluation, as in malaria parasites, this type of standardization may be sufficient. If a microscopy application demands discrimination of subtle color differences to measure concentrations or sub-cellular properties, the light source may also need to be selected to meet additional metrics for its spectral distribution – an issue of particular concern for solid state lighting solutions [42].

To avoid color-balancing issues, the white balance state must be set before image acquisition by using a consistent software preset for phones on which it is available or by imaging a reference sample illuminated by a known light source when such presets are not accessible (Figure 7C). The automatic adjustment of the white balance must be disabled during subsequent image acquisition to keep color response constant. By utilizing these calibration steps, users can capture images with color content appropriate for any downstream post-processing, analysis or decision-making steps (Figure 7D). The Digital Imaging and Communications in Medicine (DICOM) standard, which is a format for exchanging medical image data, supports storage of information about illumination conditions and the capture device [43]. Thus, a combination of a calibrated phone and light source can be used to collect images as part of a reproducible and DICOM-compliant workflow for color management.

#### Information content is altered by image processing

Once images have been collected, modern mobile phones automatically implement image processing, including image compression and edge sharpening. In contrast, scientific cameras carry out no compression, sharpening, or any other processing on image data without user instruction. Mobile phones typically apply a JPEG compression algorithm prior to storage, raising a concern about information that might be lost from and artifacts added to the resulting images. Significant work has gone into balancing image compression and file size while maintaining diagnostic value [44]–[46]. DICOM guidelines indicate that a file size reduction of 15–20x for storage can maintain diagnostic quality [43], and the high quality storage setting of most phones fulfills this requirement for compliant imaging (Figure 7E). The image of the Wright stained blood cells in Figure 2B is 1.7 MB in size when using the highest quality JPEG storage setting, which is a ∼10x reduction from the theoretical 8-bit, 3-color storage size of 15 MB for a 5 megapixel image.

Mobile phones also apply automatic processing to an image to increase its apparent sharpness [34], [47]. An example of this sharpening artifact is the apparent haloing around high contrast chrome features in a USAF target captured by an HTC Wildfire S (Figure 6G). While ringing at sharp edges can also be caused by low numerical aperture (coherent) illumination, we ruled out this possibility by quantifying the edge response of the CellScope mobile phone microscope with two different phones, the iPhone 4S and Nexus 4, as well as a configurable CMOS camera with direct access to raw image data. Intensity profiles across an edge were collected, aligned, averaged to reduce noise, and compared. The edge profile produced by the CMOS camera demonstrates the expected monotonically increasing edge associated with the diffuse incoherent light source used in the system [25]. However, the edge profile of both mobile phones deviated from a monotonic profile on both sides of the edge, with overshoots and undershoots of intensity greater than 5% of the normalized response (Figure 6H). While an approach to standardize the sharpening applied at edges to compare the underlying imaging devices is available [47], it assumes a particular symmetrical functional form to the sharpening. As profiles for the Nexus 4 and iPhone 4S demonstrate, however, the automated sharpening is neither symmetric nor consistent across mobile phones. Without applying phone-specific proprietary methods to remove the sharpening, information is lost at these sharp, high contrast transitions. Though compression could also cause ringing at edges, we do not observe the blocking effect, the most noticeable artifact of the JPEG algorithm [48], and therefore expect that the low compression ratios used during image capture have only negligible contributions to the observed edge distortions as compared to automated sharpening implemented on the mobile phone.

## Discussion

The potential of mobile phone microscopy has been widely recognized. Fully functional and easy-to-use platforms for mobile imaging will continue to be a driver of emerging biomedical, agricultural, and environmental applications. In contrast to a custom-built integrated imaging device, mobile phone-based instruments can leverage the scale of commercial manufacturing to obtain a high quality camera sensor with on-board computing, GPS tagging, and auxiliary sensors at a low cost. These hardware components are supported by a software ecosystem that provides standardized development kits, image processing libraries, application distribution channels, and rapid over-the-air updating capabilities. Successful commercial applications ranging from diagnostics to coordinating the delivery of care have demonstrated that doctors and healthcare seekers can drive a market for novel phone applications, and widespread cellular towers provide mobile phones the ability to download updated tools and transmit the collected data for off-site diagnosis. To translate this potential to medical imaging applications, however, we must first answer questions about the quality and reproducibility of data captured by a mobile imaging device.

In this study, we systematically address the issue of quantitative microscopy with a mobile phone by constructing a mobile phone microscope and evaluating the quality of images taken with a range of different mobile phones. We first characterized the resolution of the optics of the CellScope mobile phone microscope, examining the extent to which phones released in different years with different pixel counts are capable of capturing the information collected by the microscope optics. We also evaluated other optical characteristics of the system including uniformity and distortion across the field of view, as well as the linearity of the phone response to intensity. We then demonstrated the difficulty of obtaining a reproducible, spectrally accurate response across multiple samples for quantitative purposes when using the default camera functionality of the phone. Finally, we outlined a protocol to minimize variation across images to enable a workflow for capturing consistent data for quantitative applications. With the standardization and recording of assay parameters, image data can be structured according to the DICOM standard, enabling the integration of specimen data into existing hospital workflows for picture archival and pathology. For long-term or scaled up use of these systems, it will be important to note changes to manufacturers or updates to image processing software which may require corresponding re-standardization.

Beyond the basic camera functionality that comes standard with most mobile phones, current versions of the Android and iOS platforms (4.4 and 7, respectively) enable development of custom camera applications. While developers cannot (yet) arbitrarily specify gain and exposure values, they do have the ability to lock capture settings that are chosen by the phone’s automated processes. They also have the ability to lock brightness and color gain independently, as well as limited control of image manipulation after capture, enabling lossless image saving in place of traditional JPEG compression. In Figure 7, we outline the use of standardized image collection processes in combination with the control afforded by mobile phone photography applications to address many of the potential barriers to quantitative imaging with a mobile phone microscope.

Certain features of the image processing applied by the on-board software cannot be disabled given the degree of control currently provided by phone manufacturers and operating system designers. As a result, phone-specific artifacts will continue to be visible in mobile phone microscope images, such as those around large, sharp, and high contrast features like edges. These artifacts do not ultimately affect the basic morphology or color information that is useful for most diagnostic applications but still represent a difference from scientific imaging. Cooperation with phone manufacturers and operating system developers could help to eliminate these minor artifacts and accelerate development of image-based diagnostic assays through access to the image-processing pipeline. Similarly, additional software control of image capture and processing would be an advantage for point-of-care devices since some of the steps we outline to improve reproducibility could be implemented in custom software. While beneficial for screening and diagnosis applications, increased control of the camera presents real challenges – companies must be willing to support additional functions and coordinate with multiple component manufacturers, and imperfect image sensor performance could no longer be hidden behind layers of processing. With the increasing acceptance of a mobile phone as a sensing and health tool, however, an opening of the camera toolkit for biomedical, agriculture, and environmental applications would have a significant impact.

## Materials and Methods

### Mobile Phones

All mobile phones were purchased from a reseller or borrowed for testing. Camera modules were inspected visually for damage before testing. The phones tested include all iPhones released from 2007–2012: iPhone (1.9 MP), iPhone 3 G (1.9 MP), iPhone 3 GS (3 MP), iPhone 4 (5 MP), iPhone 4S (8 MP), and iPhone 5 (8 MP). We also include the older HTC G1 (3 MP), the basic HTC Wildfire S (5 MP) and Samsung Galaxy Ace (5 MP), and the newer LG Nexus 4 (8 MP) in the Android family of phones. Reference information on the performance of the optics was derived from a basic monochrome, configurable CMOS camera (Thorlabs, 1545 M) with 5.2µm pixel pitch. Paired with an achromatic 50 mm focal length lens, this yielded a sample magnification that guaranteed oversampling of a factor of at least ≥4 * Nyquist for the frequency content collected by each objective.

### CellScope Mobile Phone Microscope

The CellScope mobile phone microscopy platform consists of a finite conjugate imaging pathway built into a 3 D printed casing. Phone comparisons were done with a 10×/0.25 NA objective (Edmund Optics, #36–132) spaced 160 mm from a 20x widefield eyepiece (Edmund Optics, #39–696) with a pair of silver turning mirrors (Thorlabs, CM1-P01) to make the design more compact. The casing, stage, and phone adapters were printed using thermally extruded ABS plastic utilizing a desktop 3D printer (Dimension, uPrint Plus). Devices have been evaluated in the field with clinical partners utilizing custom holders for the iPhone 4 and HTC Wildfire S [20]. All tests for this work, however, were done with a single holder that could be adjusted to mount any of the phone types before image capture. The sample was illuminated using a keychain white-LED flashlight (Amazon, Streamlight 72001 Keymate LED Flashlight). The flashlight can be powered by field-swappable watch batteries, but for testing purposes we utilized a constant 4 V power supply attached directly to the LED. This light source is diffused using a thin sheet of ABS printed as part of the flashlight-holder that is 18 mm in diameter and 19 mm away from the sample plane. This yields an approximate NA of 0.4 based on the angle defined by the diffuser radius and distance to the sample; the diffuser and large NA condenser are important to avoid the resolution penalty and ringing artifacts associated with low numerical aperture illumination with a single bare LED. Testing additional resolution and field of view configurations involved simply switching to other achromatic objectives from Edmund Optics, e.g. 4×/0.10 NA (#36–131), 20×/0.40 NA (#38–339), 40×/0.65 NA (#36–133), and 60×/0.85 NA (#38–340).

### Spatial Resolution Measurements

To find the limiting resolution of each system, images were captured using the default camera functionality on each phone. The smallest visible groups on a high resolution 1951 USAF Air Force Target (Ready Optics, encompassing Elements 1–6 of Groups 4 to 11) were centered and aligned to match the orientation of the pixel array on the camera phone sensor. Line profiles were taken across both vertical and horizontal groups, a background subtraction was conducted, and the resolution was chosen as the spacing of the smallest set of lines for which the average peak to trough Michelson contrast across the center bar was greater than 10% in both axes. The size of the inscribed field of view was determined using a Ronchi ruling with a defined number of lines per millimeter. Since this field of view remained constant for each objective, the size of the circle could be used to calibrate a sample-referenced pixel pitch for each captured image even as the auto-focus functionality of the phone varied the apparent field size dynamically.

### Distortion, Uniformity, and Linearity Measurements

Distortion was quantified by taking a line profile across an image of a bar pattern with a constant spacing of 32 line pairs per millimeter. The positions of the centers of the lines were used to determine how the apparent spacing of the lines varied radially. The line spacing was then fit to a quadratic [26] and normalized to the center spacing to obtain the percentage distortion across the field of view. For uniformity and linearity measurements, an emission filter centered at 540 nm and pass band width of 40 nm (Chroma Technology, D540/40 m) was placed behind the objective to spectrally define the illumination source and allow us to analyze the green channel in isolation. The uniformity of the illuminated field of view was determined by bringing a sample into focus, translating to a clear field of view, and averaging line profiles across the field to determine the variation in intensity as a function of radial position. This variation was then normalized to the intensity value in the center of the field. To measure the pixel response of the mobile phone, the intensity of the diffused, spectrally defined light source was calibrated as a function of applied voltage using a fiber spectrometer (Ocean Optics, USB 650 Red Tide Spectrometer). A series of intensities was then measured using the mobile phone microscope, and the input normalized to the highest non-saturating intensity. The measurements were then transformed by a gamma factor of 2.2 [27] by normalizing the intensity by the maximum sensor output (255 for an 8-bit value), applying an exponent of 2.2, and rescaling by a factor of 255 to restore the full range. This procedure resulted in excellent sensor linearity with intensity (R^2^ value of 0.999; Figure 3C). Note that the cellphone cameras, like all other commercial cameras meant to be used with standard displays and printing technology, encode image information with a standard sRGB color profile [27]. By this standard, the intensity information is encoded with a gamma factor of 0.45 as is done by other consumer devices so that monitors with the inverse gamma factor of 2.2 can linearly display the information. It is only when the application requires analyzing the intensity of the image instead of displaying it that this gamma encoding needs to be undone (thus recovering the linear response shown in the figure).

### Image Contrast Measurements

To isolate the effects of individual capture parameters on image properties, the Almost DSLR application (iTunes) was installed on an iPhone 4 to independently control exposure time, white balance, and focal position. The 20 µm diameter pinhole array used to demonstrate saturation effects was obtained from Thorlabs (# R1L3S5P). Blood smear samples used for imaging were purchased from Carolina Biological Supply (# 313158). To elucidate the effect of focal position on feature size, the focus was locked in the near and far state in traditional photography mode. The near mode was chosen as the closest the object the phone could successfully focus on and the far mode was set by locking on an object beyond the hyperfocal distance of the phone. The exposure was locked on the same clear field of view to eliminate any color or exposure based effects. To then isolate the effect of exposure settings, the focal distance was locked to the near setting while either locking the exposure to a clear field of view or enabling the auto-exposure while acquiring images of the pinholes. Finally, the phone was allowed to adjust its white balance state while using either the LED flashlight described above or a custom assembled halogen light source to demonstrate the interplay of color gain and illumination spectrum.
